# Rapid Progression of Primary Sclerosing Cholangitis Complicated with Ulcerative Colitis

**DOI:** 10.1155/2015/125718

**Published:** 2015-01-28

**Authors:** Piotr Pardak, Ewa Walczak, Rafał S. Filip

**Affiliations:** ^1^Department of Internal Medicine, Institute of Rural Health, 20-090 Lublin, Poland; ^2^Department of Clinical Endoscopy, Institute of Rural Health, 20-090 Lublin, Poland; ^3^Department of Public Health and Social Medicine, University of Rzeszów, 35-959 Rzeszów, Poland

## Abstract

Primary sclerosing cholangitis is a cholestatic condition with unknown etiology and long-standing, progressive course, leading to cirrhosis and requiring orthotropic liver transplant. In approximately 80%, primary sclerosing cholangitis is accompanied by inflammatory bowel disease, and in most cases the recognition of bowel disease precedes the diagnosis of primary sclerosing cholangitis. We describe a case of 22-year-old male diagnosed simultaneously with primary sclerosing cholangitis and ulcerative colitis, with a medical history suggesting uncommon prior development of the liver disease. Five months after the initial diagnosis, we observed advanced lesions of bile tree due to progression of primary sclerosing cholangitis, which led to the unusually fast necessity for the orthotopic liver transplant.

## 1. Introduction

Primary sclerosing cholangitis (PSC) is a chronic cholestatic condition with unknown etiology. The outcome of the progressive fibrosis and destruction of bile ducts is a persistent cholestasis leading to the development of liver cirrhosis. It is estimated that, in approximately 80%, PSC is accompanied by inflammatory bowel disease (IBD), mostly ulcerative colitis (UC). However, UC may remain clinically silent; therefore, the course of PSC usually does not correlate with intestinal activity. In most cases, the recognition of IBD precedes the PSC diagnosis by several years. The natural history of PSC is long-standing and variable and frequently progressive, leading to cirrhosis, requiring orthotropic liver transplant (OLT). The most ominous adverse event of PSC is the development of cholangiocarcinoma (CC) [1–6].

## 2. Case Report

A 22-year-old male was referred to the Department of Internal Medicine due to periodic abdominal pain accompanied by loose stools and rectal bleeding lasting for a couple of weeks. General weakness, pruritus, and weight loss were also noticeable. He had no noteworthy medical or family history, including IBD and liver diseases; however, abnormal liver function tests were first noted approximately 1 year earlier. He had no history of drug abuse or significant alcohol consumption prior to admission. Physical examination showed grey-sallow colour of the skin, yellowness of the sclera, singular mutilations in the skin area of the abdominal cavity and thighs, and hepatomegaly. Laboratory tests showed anaemia as well as elevated levels of total bilirubin and hepatobiliary enzymes. Other clinical tests, including those for infectious diseases, were normal (Table 1).

**Figure 1:**
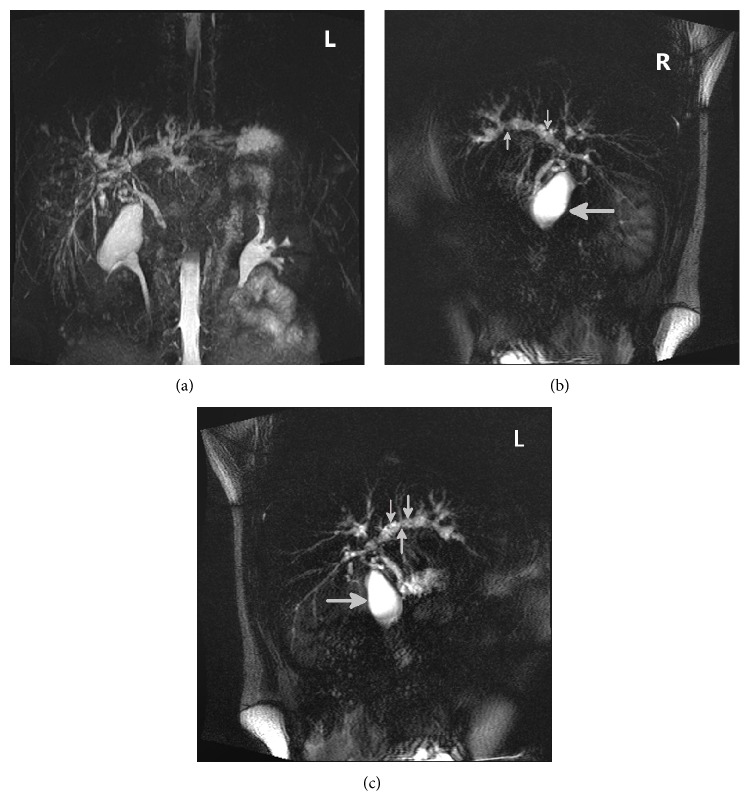
Irregular dilatations and constrictions of bile ducts (small arrows) diagnosed in MRCP (gall bladder, large arrows).

Abdominal ultrasound examination showed hepatosplenomegaly and slight dilatations of bile ducts in the area of the left liver lobe. Enhanced computerized tomography (CT) demonstrated hepatomegaly and splenomegaly, lymphadenopathy, and changes suggesting the presence of inflammation in the area of intrahepatic bile ducts. Magnetic resonance cholangiopancreatography (MRCP) revealed the presence of irregular dilatations and constrictions of the intrahepatic bile ducts (especially in the area of left liver lobe), suggesting the recognition of PSC (Figures 1(a)–1(c)).

Endoscopic examination of the large bowel showed erythema and friability of the mucosa and loss of vascular pattern in the whole colon. More intensive inflammatory changes with additional superficial erosions and ulcers were visible within the sigmoid colon (Mayo UC score: 2/3). Histology confirmed inflammatory changes of the mucosa which are characteristic for UC. The presence of the hiatus hernia and slight superficial inflammation of the antral mucosa was diagnosed during esophagogastroduodenoscopy. The patient received typical treatment with glucocorticosteroids p.o. (prednisone 30 mg/day) and mesalazine p.o. (3 g/day) and p.r. (4 g/day) with good tolerance and satisfactory clinical response.

Three months later, the patient was hospitalized due to elevation of aminotransferases, total bilirubin, alkaline phosphatase (ALP), and gamma-glutamyl transpeptidase (GGTP). Endoscopic retrograde cholangiopancreatography (ERCP) showed significant constrictions of bile ducts (intrahepatic bile ducts did not contrast virtually, only partially in the right liver lobe), a sphincterotomy was performed, and a plastic stent was implanted in the common bile duct. During hospitalization, PSC-AIH (autoimmune hepatitis) overlap syndrome was also considered; however, on the basis of all available clinical data, its probability was estimated as very low. It is important to note that abdominal ultrasound, due to parietal solid lesion of uncertain etiology in the gallbladder, raised the suspicion of malignancy, which was subsequently excluded. However, given rapidly progressing PSC which would require transplantation in the future, the patient was transferred to the hepatology referential centre for cholecystectomy. UC was in remission and none of the intestinal symptoms were present at the time.

Five months later, the patient was once more referred to the transplantology centre. The general condition of the patient was poor, and laboratory tests showed impaired liver function (with cholestasis and jaundice), additionally complicated by local symptoms suggesting bacterial inflammation. ECPW was performed, and further exacerbation of lesions characteristic for PSC combined with the inflammatory changes was seen. A common bile duct free passage was restored by the reimplantation of a new stent (the previous stent was expelled prior to admission). After completion of all the tests included in the OLT qualification protocol, the patient was enrolled on the liver recipient's waiting list.

OLT was performed three months later. Histopathological examination of excised liver showed preserved architecture of the organ and severe fibrosis in the majority of the portobiliary sinuses, with bands of connective tissue penetrating into the parenchyma and separating more clearly demarcated nodules. Within the portobiliary sinuses and in some bands of connective tissue were found mainly chronic inflammatory infiltration and multifocally devastation of the wall of the bile canaliculus. Multifocally, within the walls of large interlobular and hilum bile ducts, massive inflammatory infiltrations, almost complete disappearance of the epithelium, and numerous mucosal ulceration were found. In addition, locally, in the portobiliary sinuses exacerbated growth of neocholangioli was present. Characteristic periductal fibrosis was found around the walls of some bile ducts. Hepatocytes were without significant abnormalities; in the gallbladder, chronic inflammation was found. The above result supported the diagnosis of PSC.

The patient's postoperative course was complicated by bleeding from the vascular anastomosis approximately one month after OLT and provided with surgical treatment. The patient is well 15 months after transplantation.

## 3. Discussion

It is commonly believed that the PSC has a long-standing course; however, the dynamics of the destruction of the biliary duct system is highly variable, although usually leading to liver-related morbidity, mortality, and the need for liver transplantation [1, 4–7]. Usually, IBD symptoms precede the occurrence of PSC, even by several years, and PSC is often identified during follow-up in patients treated for IBD. The scenario in which liver symptoms appear before the development of IBD is rare [1–4, 8–13]. In the presented case of a 22-year-old male, PSC and UC were diagnosed simultaneously, although the medical data indicated prior development of the liver disease. However, the possibility that nonspecific inflammatory lesions in the colon mucosa were present without any clinical symptoms of UC cannot be excluded. Such a possibility was observed among patients with PSC combined with UC, as described later by Swedish authors [14].

Clinical symptoms of PSC usually develop together with progression of the inflammation, fibrosis, and strictures of the biliary tree [1, 4, 6]. The average time from the PSC diagnosis to liver transplant or death ranges between 9 and 18 years; however, it is strongly dependent on specific patient characteristics and the overall number of performed liver transplants. The average life span of PSC patients before liver transplant introduction was approximately 9.6–18 years and extended by OLT up to 25.5 years [5–7]. The major indications for OLT in PSC are hepatic failure, suspicion of CC, major advancement of destructive lesions in the bile ducts, and recurrent bacterial cholangitis [2, 11, 15].

Patients with PSC are at increased risk for malignancy of the gallbladder; therefore, any change within the gallbladder found on imaging studies should be an indication for an urgent cholecystectomy [16, 17].

In the presented case, 5 months after the initial diagnosis, very fast progression of bile tree destruction due to PSC was noted, with bacterial cholangitis, chronic symptoms (general weakness, pruritus), and lesion of uncertain etiology within the gallbladder, which raised the suspicion of malignancy. The coexistence of these factors with poor efficacy of the endoscopic treatment resulted in the unusually fast necessity for the OLT which, in fact, shortened the natural history of PSC in this patient to less than 12 months.

To the best of our knowledge, this is only description of a very rapid course of PSC which finally led to the OLT. However, in the cited case the OLT was performed 3 years after the initial diagnosis, mainly due to the suspicion of CC (which was subsequently confirmed) [18].

## 4. Conclusion

We describe an example of a particularly rare and fast progression of PSC, which led to the OLT in less than 12 months from establishing the initial diagnosis. Moreover, PSC was diagnosed simultaneously with UC and the patient's medical history suggested uncommon, previous to UC, development of the liver disease. This report highlights that patients with an atypical course of the PSC may benefit from early referral to the hepatology referential centre.

## Figures and Tables

**Table 1:** Laboratory tests results on PSC diagnosis and on OLT qualification.

Laboratory parameter	PSC diagnosis (02.2013)	OLT qualification (07.2013)
WBC [×10^3^/uL]	9.86	13.5
RBC [×10^6^/uL]	4.24	4.8
HGB [g/dL]	10.3	14.1
PLT [×10^3^/uL]	342	287
ALP [U/L]	1,097	354
GGTP [U/L]	677	378
Bil. tot. [mg/dL]	1.6	1.0
ALT [U/L]	104	38
AST [U/L]	50	22
Albumin [g/dL]	3.4	4.1
INR	1.04	0.9
Fibrinogen [g/L]	472	443

WBC: white blood cells, RBC: red blood cells, HGB: haemoglobin, and INR: International Normalized Ratio.
